# Interventions for Improving Leisure for Older Adults on the Palliative Pathway Living With Advanced Cancer: A Qualitative Systematic Review

**DOI:** 10.1177/03080226251367641

**Published:** 2025-10-27

**Authors:** Collette Crilly, Sureshkumar Kamalakannan

**Affiliations:** Department of Social Work, Education and Community Wellbeing, Northumbria University, Newcastle Upon Tyne, UK

**Keywords:** Occupational therapy, leisure, palliative care, older adults, qualitative methods

## Abstract

**Introduction:**

While occupational therapists (OTs) promote quality of life through meaningful occupation, little attention has been paid to the role that leisure plays in palliative care. This review aims to explore the perceptions of service users and OTs regarding engagement in leisure activities at the end-of-life.

**Objective:**

1. Determine the value of leisure activities for older adults living with cancer on the palliative pathway, 2. To identify interventions to promote occupational engagement.

**Method:**

A qualitative systematic review was conducted in accordance with the PRISMA Statement. The electronic databases namely ASSIA, AMED, CINAHL, Medline, PsycArticles, and PubMed were searched. Qualitative studies meeting the eligibility criteria were included. Two reviewers independently screened the identified articles. The findings were qualitatively synthesised using thematic analysis.

**Results:**

We included seven articles with 405 participants. This review demonstrated that leisure could help people in palliative care maintain a sense of identity and normalcy. Leisure also increased happiness and provided relief from pain. Occupational therapy interventions targeting leisure allowed people to maintain a consistent level of engagement.

**Conclusion:**

Further empirical research is required to explore palliative patients’ perspectives on leisure and to develop specific occupational therapy interventions that enable OTs to facilitate leisure engagement for people in palliative care.

## Plain Language Summary

Why we conducted this review:

• There is little focus on engaging people in leisure when they experience palliative care and there is a big emphasis on managing symptoms of cancer.

• More and more people need palliative care due to the increasing ageing population who live longer with debilitating chronic illness.

**What did We do**:

• We systematically identified relevant articles and reviewed the literature from the past 14 years to understand the perspectives of older adults receiving palliative care on participating in leisure.

What did we find out:

• Older adults living with cancer value leisure activities because leisure gives them a greater sense of control over their lives, helps them to maintain a sense of normalcy, provides relief from pain, and increases feelings of happiness.

• Occupational therapists have the skills to enable people to participate in leisure by changing the way an activity is done or changing the environment.

## Background

Palliative care is a method of improving the quality of life of people and their families who are dealing with the effects of terminal illness. Palliative care specialists provide symptom management, emotional, spiritual, and psychological support, as well as practical assistance such as equipment and advanced care planning to patients and their families (National Cancer Institute, 2021). Consequently, palliative care can help people live longer and more comfortably, and achieve a ‘good death’, which is the foundation of government policy, such as the Department of Health’s End-of-Life Care Strategy (2008; Department of Health, 2008, p. 12).

An estimated ‘56.8 million people’, including ‘25.7 million’ in their final year of life, require palliative care, with the majority living in low- and middle-income countries (LMICs; World Health Organisation—WHO, 2020). However, only about ‘14% of people’ in the world who require palliative care receive it (WHO, 2020). In the United Kingdom (UK), ‘85%’ of people who die are ‘65 or older’ (Age UK, 2019, p. 1), and it is predicted that by 2050, one in every four people will be 65 or older (Morgan, 2019, p. 2). As a result, the global demand for palliative care is expected to rise from ‘25% to 47%’ by 2040 as people live longer with chronic long-term conditions (Dudley & Mutebi, 2022, p. 1).

Palliative care is required for a variety of chronic conditions such as heart failure, dementia, chronic obstructive pulmonary disease and Parkinson’s disease (Gómez-Batiste et al., 2017). The focus has primarily been on improving quality of life, symptom management and implementing effective care plans for these life-limiting chronic conditions (Kircher et al., 2020). Palliative care, as often understood, is particularly important for people living with cancer (National Cancer Institute [NCI], 2021). A cancer that is not likely to respond to treatment and is therefore likely to progress is defined as advanced cancer (NCI, 2023). The most common and serious symptoms are pain, fatigue, and shortness of breath, with ‘80%’ of cancer patients experiencing moderate to severe pain (WHO, 2020). While using opioids to relieve pain and respect a person’s dignity is an ethical responsibility, these are not the only factors that are important to palliative patients (WHO, 2020).

According to Shallwani et al. (2023), people living with cancer also experience a decline in physical function, resulting in significant occupational disruption and deprivation due to the unpredictable physical, psychological, and emotional symptoms of cancer. This causes unpleasant emotions, powerlessness, loss of dignity, and psychological pain (Badger et al., 2016). Occupational therapists (OTs) in palliative care break down people’s occupations into component parts to understand the person, task, and environmental demands to adapt and grade them to facilitate engagement and improve performance (Butler & Henry, 2011). This is significant because people wish to maintain occupational roles and to participate in meaningful occupations as much as possible until death (Bentz et al., 2021).

Engaging in leisure activities at the end-of-life leads to increased energy and happiness, a sense of flow, relief from pain and negative emotions, improved communication and comprehension of one’s feelings, and a stronger sense of self-identity (Reynolds & Prior, 2006). However, little attention has been paid to the role that leisure plays in palliative care, with the focus more traditionally on the medical model that encourages healthcare professionals to prioritise symptom management and control with little regard for occupational engagement, resulting in role loss and occupational alienation for older adults living with cancer (Al-Abdin et al., 2020). This is evident in Rome et al. (2011) review, which found that psychosocial needs, including leisure, were the most prevalent unmet need in palliative care.

Several factors limit patients’ access to leisure activities in palliative care, including a lack of evidence for providing non-pharmacological interventions (Tavemark et al., 2019). Hence, there is a lack of awareness and understanding of the role of occupational therapy in palliative care. Patients and medical professionals are unaware of the unique role OTs play in facilitating leisure participation (Keesing & Rosenwax, 2011). This, combined with time and financial constraints, means that health professionals must choose which interventions to implement while keeping costs low; thus, leisure is not addressed (Tavemark et al., 2019). Hence, a qualitative systematic scoping review of the current evidence base is required to understand the impact of leisure on occupational engagement of those receiving palliative care and its implications for practice, policy, and future research.

## Aims and Objectives

This review aims to explore the perceptions of palliative patients, informal carers, and Occupational therapists on engaging in leisure activities at the end-of-life.

## Objectives

The primary objective of this review is to systematically review relevant literature to:

1. Understand the perspectives of cancer patients and OTs concerning the value of leisure.
2. To identify interventions that promote occupational engagement and examine their benefits.

## Method

### Criteria for Considering Studies for This Review

#### Types of Participants

Participants aged 65 years and over living with advanced cancer on the palliative pathway in a hospice setting, hospital or at home were included. We also included studies that focused on OTs, formal or paid caregivers and informal carers such as family members, relatives or friends working in palliative care.

#### Type of Intervention

The intervention is low-intensity leisure activities that are provided in palliative settings, at home or in hospital. The reviewers included leisure as an overarching term in the search and narrowed leisure down to low-intensity leisure that could be performed while sedentary and was not physically demanding to increase the likelihood of engagement.

#### Types of Outcome Measures

The outcome measure was perceptions of leisure engagement among palliative patients, as well as any outcomes that measured the effectiveness of interventions to promote leisure engagement at the end-of-life.

#### Type of Studies

In this review, all qualitative studies were included. We also included mixed methods studies that obtained qualitative data related to the review objectives. These studies used semi-structured interviews and diary entries. Quantitative studies such as cross-sectional, case control, and randomised controlled trials were excluded.

### Search Methods for Identification of Studies

#### Electronic Searches

A scoping search was conducted by two reviewers to aid in the formulation of the research question and the identification of key search terms. The research question for this review was explicitly defined by dividing the question according to the patients, interventions, comparisons, outcomes, and study design (PICOS) model. Given the lack of extensive research on this topic, particularly in occupational therapy, the reviewers aimed to not miss any articles related to this review. Hence the PICOS model was chosen to develop a broad search strategy with all relevant search concepts. The PICOS model was also used to structure the review question because it ensures that the key elements of the question are well defined (Methley et al., 2014). We did not have the comparison or the C component of the PICOS in our search, as this was not relevant to this qualitative review. The results were limited to the English language, owing to the research staff members’ timelines and translation resources. Please find Table 1 describing the PICOS concepts used in building the search strategy. The electronic databases searched were ASSIA, AMED, CINAHL, Medline, Psych Articles, and PubMed. A combination of health-related, social care, and nursing databases were searched to produce a systematic search and ensure that all relevant articles were captured to answer the research question, thereby minimising bias (Soilemezi & Linceviciute, 2018).

**Table 1.** Search string.

Picos elements	Keywords	Search terms	Search string
P (Patient or population)	Palliative patients aged 65 years and over in a hospice setting, hospital or living at home. Family members who are informal carers of relatives aged 65 years and over on the palliative pathway Occupational therapists working with patients aged 65 years and over on the palliative pathway	Palliative Care Elderly Occupational Therapy	‘Palliative Care’ OR ‘End of Life’ OR ‘Hospice Care’ ‘Occupational Therapy’ AND ‘Occupational Therapist’
I (Intervention)	Low-intensity leisure activities	Leisure Recreation	‘Leisure’ OR ‘Recreation’
C (Comparison)	No comparison	No comparison	No comparison
O (Outcome)	Perceptions of palliative patients and informal carers, and occupational therapists	Perceptions Perspectives Experiences Lived experiences Barriers Enhancers Feelings Values	‘Perceptions’ OR ‘Perspectives’ OR ‘Experiences’ OR ‘Lived Experiences’ OR ‘Barriers’ OR ‘Enhancers’ OR ‘Feelings’ OR ‘Values’
S (Review Design)	Qualitative studies	Qualitative In-depth interviews Focus group discussions	‘Qualitative’ OR ‘In-depth interviews’ OR ‘Focus group discussions’

University librarians were consulted to refine the search strategy and combine key terms with free-text words. The reviewers added expanders to broaden the scope of the search by including words related to the keywords to locate studies that might otherwise have been missed. The search terms were combined using Boolean operators, which allow search terms to be combined in specific ways to broaden or narrow the search and exclude unwanted concepts that do not meet the inclusion criteria (University of Michigan Library, 2023).

Limiters were applied to the search to narrow the results to the most relevant content, and care was taken when applying limits to ensure relevant articles were not lost inadvertently (University of Michigan Library, 2023). The search strategy was limited to the years 2008 to 2022 because the End-of-Life Care Strategy (2008) was published, which was the first comprehensive framework aimed at promoting high-quality care across the UK for all adults approaching the end-of-life (Department of Health, 2008). Consequently, the decade that followed was crucial in understanding the quality of palliative care.

It was an iterative process to find the correct balance between a comprehensive search and a sensitive search. The authors increased the sensitivity of the search by modifying key words based on what had already been retrieved and using synonyms for each concept. The precision of the search was increased by clearly defining the research question and objectives and using Boolean operators to combine concepts (University of Michigan Library, 2023). The reviewers limited the articles to peer-reviewed journals because this is integral to ensuring that robust and high-quality research is published due to critical examination by recognised experts in the field (Lasserson et al., 2023). Therefore, grey literature was not included due to the absence of peer review of unpublished literature.

#### Searching Other Resources

To ensure that no relevant studies were missed, both authors conducted supplemental searches of alternative sources such as Google Search, as well as perused the reference lists of identified articles to examine and identify further relevant studies. See Table 1 for the PICOS-based search string, which includes key words, search terms, and Boolean operators.

### Data Collection, Management and Synthesis

#### Selection of Studies

Two authors independently screened the study titles for inclusion. Duplicates were identified and removed. Both reviewers assessed the titles and abstracts of the identified studies to establish eligibility and discarded those that were irrelevant. Secondly, both reviewers obtained the full text of all studies identified as possibly relevant and assessed them for inclusion in the review.

#### Data Extraction

Both reviewers extracted data independently using a Microsoft Excel Spreadsheet because it provided greater flexibility in what data to collect (University of Canberra, 2022). The spreadsheet was created with the Cochrane Data Extraction form as a guide (Higgins et al., 2022). Using thematic analysis, the data extraction form was used to extract key themes relevant to the review objective (Nowell et al., 2017). The form also extracted information about the study’s title, publication date, country of study, demographic information, methodological details, limitations, and recommendations.

#### Assessment of Methodological Quality

The Mixed Methods Appraisal Tool (MMAT) was used (Hong et al., 2021) to appraise the methodological quality of the included studies. This tool has been chosen because the included studies use a combination of qualitative methods and research designs, and the MMAT is efficient in allowing for the simultaneous evaluation of multiple types of research (Hong et al., 2021). The authors independently conducted the assessment of methodological quality of the included studies. Any inconsistencies were resolved through discussion; if unresolved, an expert qualitative researcher was available to resolve disagreements. Authors reported the methodological quality of included studies using a MMAT Table 4 as well as a narrative description of the quality of included studies.

#### Data Management and Synthesis

Thematic analysis was applied to assist both reviewers in detecting similarities and differences between the varying perspectives of participants to establish key themes (Nowell et al., 2017). The authors independently went beyond the content of the original studies by considering the themes against the original review question. Once initial interpretations were obtained, both reviewers developed analytical themes and subthemes.

#### Review Author Reflexivity

Both authors demonstrated reflexivity by holding moderation meetings every few weeks to reflect on review findings and negotiate research process decisions. Recognising the authors’ personal biases and influences on the research findings improves rigour because it acknowledges the researchers’ professional background, research areas of interest, and the impact these positions have on all stages of this review (Olmos-Vega et al., 2023). Beyond the author reflexivity, the authors had identified an expert OT who will resolve any inconsistencies and disagreements between them during the entire review process, particularly data extraction, synthesis, thematic analysis and assessment of methodological quality.

## Results/Findings

**Figure 1.**
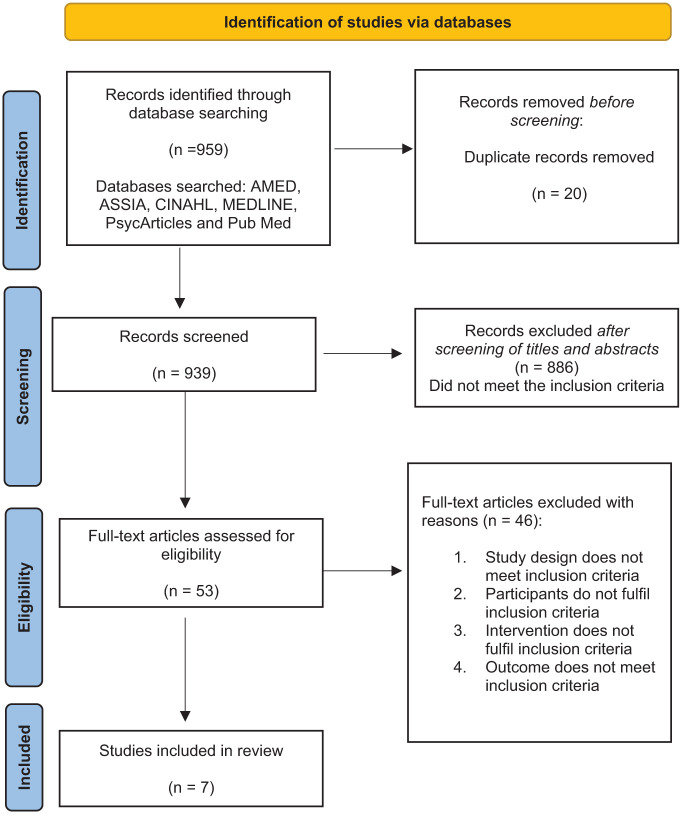
PRISMA flow diagram. *Note.* PRISMA=Preferred Reporting Items for Systematic reviews and Meta-Analyses. *Source.* Page et al. (2021).

The Preferred Reporting Items for Systematic reviews and Meta-Analyses Statement (PRISMA, 2020) was used to guide the conduct and reporting of searches (PRISMA, 2023). The search yielded 959 records via electronic databases. After deduplication, 939 studies were eligible for screening. After screening for eligibility, 53 articles were identified as eligible for full-text screening. 46 of these studies did not meet the inclusion criteria. In this review, seven studies were eligible for inclusion. A modified PRISMA flow diagram of the process of study selection is presented in Figure 1.

## Review Characteristics

### Description of Studies

Seven studies met the inclusion criteria with a total of 405 participants across all studies. The time of studies ranged from 2008 to 2022. Four studies were qualitative mixed methods studies (Lyons et al., 2013; Imanishi et al., 2015; Hammill et al., 2019; Talbot-Coulombe et al., 2022), and three studies were qualitative studies (la Cour et al., 2009; Keesing & Rosenwax, 2011; Peoples et al., 2017).

Participants included OTs (*n* = 229) working with terminally ill patients, informal carers (*n* = 14) acting as proxies for bereaved family members, and people living with advanced cancer (*n* = 162). Participants’ ages ranged from 24 to 91 years. The studies were all conducted in high-income countries; two were conducted in Australia, two in Denmark, one in Japan, one in the United States, and one in Canada. Review participants were recruited via face-to-face invitation to participate during consultation with healthcare professionals, by research staff through weekly collaboration with oncology providers in a few studies, and through follow-up clinic visits. Participants were also recruited via email invitations and letters to participate, as well as via health service newsletters, support groups, volunteer agency flyers and community newspapers. See Table 2 below for details on characteristics of included studies.

**Table 2.** Description of studies included in the review.

Date	Country	Type of patients	Age (years)	Study design	Data collection	Interventions	Outcomes measured
2019	Australia	Occupational Therapists currently treating or have treated patients with terminal cancer within the past 3 years	No information	Mixed methods survey	Combination of open-ended and close-ended questions using web-platform Survey Monkey	Everyday occupations that are prioritised	Occupational therapist’s perceptions on patients’ occupational engagement post-diagnosis of advanced cancer
2015	Japan	Stage 4 Lung Cancer with metastasis	66	Qualitative ethnographic study	Ethnographic qualitative survey	Occupational therapy intervention	The PGC Morale Scale and 100-Point Satisfaction Scale were utilised to measure quality of life
2011	Australia	Informal carers (as proxies for relatives who had died) and occupational therapists with palliative patients on caseload	No information	Mixed methods study	Email survey and semi-structured interviews	Daily experiences and occupational needs during the palliative phase	Occupational therapists’ perceptions of current scope of practice in palliative care and carers perspectives of occupations engaged in at the end- of-life
2008	Denmark	Lung, breast or colon cancer	39–80	Qualitative study	Diary writing and qualitative interviews	Everyday activities or occupations of people with advanced cancer	Patients’ perceptions of patterns of engagement over time, and preferred occupations to engage in, and participant’s level of comfort and state of mind for these activities
2013	United States of America	Breast, gastrointestinal, hematologic, genitourinary, lung, head or neck cancer	60 years and older	Mixed methods study	The Activity Card Sort (ACS) and semi-structured interviews	Occupational patterns and perceptions	The ACS was developed to measure the levels of engagement in instrumental, leisure and social activity levels of older adults with advanced cancer
2017	Denmark	Lung, colon, prostate, cervix, breast or other types of cancer not specified	18–89	Qualitative descriptive study	Semi-structured interviews	Sedentary indoor occupations	Perspectives of patients living with advanced cancer on occupational engagement and participation
2022	Canada	Occupational therapists working with patients with terminal cancer	24–60	Mixed-methods study	Online survey comprising of open-ended and close-ended questions	Mobility transfers and hygiene were prioritised as meaningful occupations to optimise comfort and safety	Occupational therapists’ perspectives of the current scope of practice and barriers, as well as education on palliative care

*Note.* ACS = Activity Card Sort; PGC *=* Philadelphia Geriatric Center.

### Review Findings

The qualitative analysis of studies in the review led to five overarching themes, including two subthemes from the data. These included (a) Perspectives of patients and OTs about leisure and recovery. (b) Occupations performed by the patients. (c) Occupational performance and engagement. (d) Interventions to promote engagement. (e) Impact of interventions to promote engagement.

Five studies described leisure occupations (Hammill et al., 2019; Keesing & Rosenwax, 2011; la Cour et al., 2009; Lyons et al., 2013; Peoples et al., 2017). Five studies explored leisure engagement and performance in cancer patients (Hammill et al., 2019; Keesing & Rosenwax, 2011; la Cour et al., 2009; Lyons et al., 2013; Peoples et al., 2017), and all studies discussed interventions to promote engagement, as well as their impact. Three studies looked at OTs’ perspectives on patients’ leisure participation (Hammill et al., 2019; Keesing & Rosenwax, 2011; Talbot-Coulombe et al., 2022), and four studies explored patients’ perceptions of leisure participation (Imanishi et al., 2015; la Cour et al., 2009; Lyons et al., 2013; Peoples et al., 2017). See Table 3 for results from the thematic analysis.

**Table 3.** Results from the qualitative thematic analysis.

Theme	Subtheme	Synopsis
Perspectives of patients and occupational therapists about leisure and recovery	Occupational therapists Patients	Occupational therapists voiced barriers to practice including a misunderstanding and reductionist view of their role as merely equipment prescribers, and the impact of staff shortages, lack of funding and excessive workloads on occupation-centred practice. Patients expressed their occupational priorities changing as they neared the end of their life to focus on leisure and social participation rather than their illness to improve quality of life, and described the negative feelings associated with loss of occupations and changes in occupational engagement.
Occupations performed by the patients		These occupations included gardening, watching TV, cooking, fishing, reading. Creative leisure occupations were prioritised, such as making photo albums, writing letters, cards and autobiographies. Some participants as well as medical staff focused on occupational goals centred around self-care, sleep, and increasing comfort and safety in transfers, mobility, feeding and hygiene. Other participants prioritised occupations that were associated with preparing for death.
Occupational performance and engagement		Occupational performance fluctuated due to the physical effects of cancer, discomfort and fatigue, leading to reduced engagement. Therefore, participants moved on a continuum between focusing on living and preparing for death. Some saw decreased engagement in a positive light and as a natural progression; however, for others disruption to occupations, loss of roles, habits and routines affected people’s sense of identity.
Interventions to promote engagement		Interventions to promote engagement included a re-focusing of occupations to focus on social participation as palliative patients wanted to engage in activities with others compared to non-palliative patients. Grading and adapting of occupations were another intervention to enable individuals to maintain partial independence which was deemed as important as full independence. Peer support groups were deemed a useful resource to engage in conversation about shared experiences. Equipment and energy conservation techniques were viewed as compensating for loss/reduced function and improved independence and reduced risk.
Impact of interventions to promote engagements		These interventions provided patients with a greater sense of control and improved quality of life as they could reprioritise engagement towards occupations that mattered most, and enabled patients to engage in daily occupations, roles and routines despite the physical effects of cancer and realisation that life is precious as patients near dying.

### Theme 1: Perspectives of Patients and OTs About Leisure and Recovery

Five studies reported on this theme. A recurring theme in the data was the importance of leisure for palliative patients in terms of contributing to improved health and wellbeing, as well as how they felt when their occupations were disrupted or lost because of cancer and treatment. Palliative patients expressed a desire to ‘spend time with their families’ and ‘leave good memories’, giving them a different focus in life which was enriching; however, did not wish to be a burden despite their illness (la Cour et al., 2009, p. 157). Creative leisure activities provoked new ways of thinking and offered creative challenge, which was motivating for patients and provided moments of flow where they could be fully absorbed in an experience that differs to the monotony of daily life (la Cour et al., 2009).

Participants accepted that recovery was a slow process; however, they struggled to adapt to their new sedentary life, which was a stark contrast to their very active life before cancer, and seeing their partners do occupations that were once their role proved emotionally challenging (Peoples et al., 2017). Participants felt that the loss of occupations made them feel that they had a boring life upon reflection, and this prompted a realisation of how different their lives now were (Peoples et al., 2017).

### Perspectives of OTs About Leisure and Recovery

Another prominent theme was OTs’ perspectives on leisure engagement for palliative patients and the barriers to facilitating engagement. OTs felt that clients’ reprioritisation of roles at the end-of-life related to valued roles that defined them as a person and contributed to them maintaining a sense of identity and normalcy (Lyons et al., 2013). OTs voiced their concerns that their role was reduced to being an equipment provider due to a misunderstanding of the role, leading to inappropriate referrals, and issues such as staff shortages meant insufficient time to meet patient’s occupational needs, creating waiting lists and a sense of not being able to do the job properly (Talbot-Coulombe et al., 2022). This is in combination with a lack of resources and excessive workloads, meaning little to no time to focus on meaningful occupation (Keesing & Rosenwax, 2011).

### Theme 2: Occupations Performed by the Patients

Two studies reported on this theme. Emerging from the thematic analysis, meaningful occupations prioritised in the palliative phase pertained predominantly to leisure with a focus on social participation. Occupations were mostly performed at home and were sedentary in nature; however, some participants expressed wishing ‘to spend time away from the home’ but that their ‘illness kept them at home’ (la Cour et al., 2009, p. 157). These occupations included gardening, watching TV, cooking, fishing, and reading. Creative leisure occupations such as making photo albums, writing letters, cards, and autobiographies were deemed an important occupation, and these items could be gifted to future generations, leaving a tangible memory of them behind; this aided in facilitating a sense of closure.

However, some participants and medical staff concentrated on occupational goals centred on self-care, such as sleep, and increasing comfort and safety in mobility, feeding, and hygiene. Other participants prioritised occupations that were associated with preparing for death. Participants stated, ‘completion of tasks to make life easier for families post death, having affairs in order, such as writing wills and organising finances’ (Hammill et al., 2019, p. 149). Participants stated that meaningful occupations should not be assumed for people living with terminal illness, but therapists should engage in open and honest narratives led by the patient to allow them to prioritise occupations prior to death ‘in their most natural environment and within the privacy of their normal lives’ (Hammill et al., 2019, p.149).

### Theme 3: Occupational Performance and Engagement

Two studies reported on this theme. Another recurring theme in the data was the impact of terminal illness on occupational engagement and performance, which appeared to worsen as cancer progressed, or because of treatment. Occupational performance fluctuated due to the physical effects of cancer, causing discomfort and fatigue leading to reduced engagement. Therefore, participants moved on a continuum between ‘focusing on living’ and ‘preparing for death’ (Hammill et al., 2019, p. 147).

Shortness of breath, fatigue and impaired balance and mobility affected functional ability; thus, daily routines and progression of their cancer influenced patients’ perception of a ‘normal everyday life’ (Peoples et al., 2017, p. 60). To increase occupational engagement, patients engaged in sedentary leisure occupations that were not physically demanding and relied on others to support with engagement. However, decreased engagement for some participants negatively affected their sense of identity, and surrendering these roles led to feelings of inadequacy and dependence. A participant who had previously lived an active lifestyle that included ‘playing tennis’ and ‘cycling’ described a ‘very boring’ schedule that consisted of mostly resting because of fatigue (Peoples et al., 2017, p. 60). Nevertheless, some saw decreased engagement in a positive light as it meant they could slow down to socialise or do more creative activities. Many participants did not express regret over reduced occupational engagement as they viewed it as a natural consequence of cancer.

### Theme 4: Interventions to Promote Engagement

Five studies reported on this theme. Another prominent theme was interventions to promote engagement in leisure occupations such as re-prioritising of occupations, grading and adapting, peer support groups, and equipment. One intervention is adapting and grading of occupations, one therapist in an included study commented that ‘it is not that the occupation itself becomes unique, but it may be the way that occupations are done that is unique, based on the person’s abilities’ (Hammill et al., 2014, p. 149). The extent to which an activity was adapted or graded was seen to be determined by a patient’s level of dependence and helped patients to adjust their expectations for what is reasonable or necessary (Hammill et al., 2014, p. 149).

Participants discussed not expecting to ‘keep the house as clean as they once did’, or socialising as much, or maintaining ‘as big a garden’ (Lyons et al., 2013, p. 36). One participant from a study stated, ‘I do one thing and then take a break, there are days where I exceed my limit and then I collapse and lie in bed for three days’ (Peoples et al., 2017, p. 61). Patients adjusted the timing or pace of an activity, eliminated steps, did activities alone, or reduced the extent to which they did them. This may be hoovering half the house one day and the other half the next or engaging in the occupation in a different way such as watching the tennis on TV, rather than playing tennis (Lyons et al., 2013). Equipment such as ‘wheelchairs’ and ‘walkers’ compensated for loss/reduced function, and improved independence in leisure participation (Talbot-Coulombe et al., 2022, p. 204).

Patients were more likely to engage in leisure activities if they did so with others. One participant from an included study stated, ‘I have lots of things I can occupy myself with, but my need is also to get together with others around craftwork, something practical like gardening’ (la Cour et al., 2009, p. 157). A carer in Peoples et al. (2017) study identified peer support groups as being potentially useful to support with engagement so that patients can confide in those experiencing the same symptoms.

### Theme 5: Impact of Interventions to Promote Engagement

Three studies reported on this theme. Interventions to improve engagement gave patients a greater sense of control and improved quality of life as they could reprioritise engagement towards most meaningful occupations. By focusing on familiar occupations, patients ‘felt alive and not like they are waiting to die’ (Hammill et al., 2019, p. 148). By maintaining some form of engagement, participants maintained their dignity and felt gratitude for completing tasks independently (Imanishi et al., 2015). However, therapists discussed the importance of ‘being, thinking, processing’ (Hammill et al., 2019, p. 149). Patients do not have to be actively ‘doing’ leisure to gain a sense of engagement (Hammill et al., 2019, p. 149). As identified in Hammill et al. (2019, p. 149) study, participants wished to sing and dance; however, if they felt physically unable, watching others perform in traditional ceremonies gave patients permission to surrender physical independence, yet remain engaged in their chosen occupations.

Adapting and grading of occupations allowed clients to ‘get more out of what they wanted to do’ (Hammill et al., 2019, p. 149). Patients found comfort in the fact that they did not have to give up activities with a new life after cancer but simply adjust what they do or the extent to which they do it (Lyons et al., 2013, p. 36). Equipment provision enhanced social participation, improved comfort, and reduced pain (Hammill et al., 2019). However, whilst social participation improved engagement in occupations as it allowed a safe space for shared experiences of living with advanced cancer, some expressed concerns in relation to too much talk about illness during activities, leading to a negative mindset (la Cour et al., 2009). Therefore, there is a need to engage in shared activity rather than shared conversation about focusing on disease and problems.

### Methodological Quality

The MMAT (Hong et al., 2021) was applied to assess the methodological quality of studies. All studies used qualitative data collection methods that were appropriate for answering the research question. The findings were accurately derived from the data in 86% of the studies, as evidenced by verbatim quotes from participants throughout. The differences and inconsistencies between qualitative and quantitative data were not adequately addressed in 67% of mixed methods studies, reducing the validity of the findings. The sampling type was not explicitly stated in 43% of the studies, introducing sampling bias as the sample may be based on convenience rather than equal probability, affecting the validity of the findings as the results cannot be generalised to the population (Shringarpure & Xing, 2014). Table 4 provides the quality appraisal carried out for the included studies indicating the percentage of methodological quality assessed for each item of the MMAT.

**Table 4.** Methodological quality of included studies (MMAT scores).

Included Studies	Lyons et al. (2013)	Imanishi et al. (2015)	Keesing and Rosenwax (2011)	Peoples et al. (2017)	la Cour et al. (2009)	Talbot-Coulombe et al. (2022)	Hammill et al. (2019)
Are there clear research questions?	YES	NO	YES	YES	YES	YES	YES
Do the collected data allow to address the research questions?	YES	CAN’T TELL	YES	YES	YES	YES	YES
*Qualitative Studies* Is the qualitative approach appropriate to answer the research question?	YES	YES	YES	YES	YES	YES	YES
Are the qualitative data collection methods adequate to address the research question?	YES	NO	YES	YES	YES	YES	YES
Are the findings adequately derived from the data?	YES	NO	YES	YES	YES	YES	YES
Is the interpretation of results sufficiently substantiated by data?	YES	NO	YES	YES	YES	YES	YES
Is there coherence between qualitative data sources, collection, analysis and interpretation?	**YES**	**YES**	**YES**	**YES**	**YES**	**YES**	**YES**
*Mixed-methods studies* 5.1. Is there an adequate rationale for using a mixed methods design to address the research question?	YES	N/A	NO	N/A	N/A	NO	N/A
5.2. Are the different components of the study effectively integrated to answer the research question?	YES	N/A	NO	N/A	N/A	YES	N/A
5.3. Are the outputs of the integration of qualitative and quantitative components adequately interpreted?	YES	N/A	YES	N/A	N/A	NO	N/A
5.4. Are divergences and inconsistencies between quantitative and qualitative results adequately addressed?	YES	N/A	NO	N/A	N/A	NO	N/A
5.5. Do the different components of the study adhere to the quality criteria of each tradition of the methods involved?	CAN’T TELL	N/A	YES	N/A	N/A	YES	N/A
Total score	11/12 (92%)	2/7 (29%)	9/12 (75%)	7/7 (100%)	7/7 (100%)	9/12 (75%)	7/7 (100%)

## Discussion and Implications

### Summary of Results

The review search identified 959 records, and we screened to identify 53 records for full-text screening. Full-text screening of 53 articles enabled us to identify 7 articles for the review. We reviewed 7 articles between the years 2008 and 2022 that met the criteria to be included in the review. The findings of this review indicate that palliative patients value leisure to contribute to health and well-being at the end-of-life. The review also found that interventions such as grading and adapting, energy conversation techniques, and equipment can facilitate engagement when participation and performance decline due to cancer symptoms and treatment.

### Findings in Relation to Existing Literature

The themes clearly indicate that participants who engaged in a variety of leisure activities that did not require much physical exertion but included a creative component. This was important in providing mental stimulation for people and something novel that differed from the monotony of everyday life, and creative activities allowed participants to become fully immersed in a task and gain a sense of ‘flow’. Participants expressed a desire to engage in leisure activities with others, which alleviated the loneliness of living with terminal illness, and patients felt as though they belonged, which was a novel finding. This was evident in a recent study conducted in Denmark, where people living alone had difficulties prioritising leisure and social interaction as opposed to those individuals with cancer who were not alone prioritising social interaction (Blichfeldt et al., 2023). According to Wilcock, this sense of belonging encourages engagement because people benefit from friendship, a sense of inclusion, mutual support, and peer affirmation (Hitch et al., 2014). However, a need to engage in shared activity rather than shared conversation about illness was identified throughout to avoid negative mindsets and a positive attitude towards engagement in leisure even with a life limiting illness.

A third theme was interventions to maintain engagement. These interventions included grading and adapting of occupations and the environment, re-prioritisation of the most important leisure occupations, equipment provision to compensate for loss of or reduced function, and peer support groups. This theme has implications not just for individuals affected by cancer but for any life-limiting chronic conditions that require palliative care (Gómez-Batiste et al., 2017). It also implies the benefits of occupational therapy that are still researched and explored to understand the strategies to enable social participation in those experiencing life-limiting illnesses (Kircher et al., 2020). Another common theme was the effect of these interventions on participants. These interventions gave people a sense of comfort in knowing that they didn’t have to give up their leisure occupations, but could simply adjust them to meet their needs, and that they didn’t have to be actively doing to stay engaged in an occupation but could gain the same sense of accomplishment by simply watching others engage in leisure. These findings support available evidence showing leisure can provide patients with a sense of normalcy, lead to a stronger sense of self-worth, and increase vitality and pleasure (Reynolds & Prior, 2006). Finally, the findings indicate that there are a few financial and service constraints that prevent or limit OTs from engaging patients in leisure activities and, hence, they focus on cost-effective interventions at the expense of patient need. This is exacerbated by a misunderstanding of the occupational therapy role in the wider team, particularly in acute settings, which makes occupation-centred practice difficult because their role is frequently reduced to equipment prescriber, resulting in professionals experiencing reduced job satisfaction and a sense that they cannot do the job properly. This is consistent with current literature, which states that leisure is the most common unmet occupational need in palliative care (Rome et al., 2011), and pharmacological interventions are prioritised over non-pharmacological interventions such as occupational therapy (Tavemark et al., 2019).

### Strengths and Limitations

The studies provided rich qualitative data to provide comprehensive insights into the phenomenon of interest through the inclusion of verbatim quotes to illuminate participants’ perspectives. This allowed the reviewers to make judgements about whether the final themes are true to participants’ accounts, increasing the validity of the studies (Noble & Smith, 2015). A mixed methods methodology allowed qualitative and quantitative data to be triangulated with one another, resulting in a more complete picture of the phenomenon as the results were cross-checked to verify the truth value of findings and increase their trustworthiness (Carter et al., 2014). However, the included studies were all conducted in high-income countries; and therefore, the findings are not representative of those living in low-and-middle income countries and should be interpreted with caution. The studies were English only, which introduced language bias, and excluded potentially relevant papers, impacting on the validity of the results (Konno et al., 2020, p. 6374). Further research is required to evaluate the impact of restricting inclusion criteria to English-only publications, as there is a wealth of research produced globally and available in all languages.

The mixed methods studies lacked a clear rationale for the review design, which is important for the reviewers to understand the added value of mixed methods and to increase confidence in the findings. This has been identified as a common problem across the literature because it requires more resources, time, and skill, and is recommended that researchers receive intensive mixed methods training to ensure that they use it appropriately to provide plausible and credible findings (Wasti et al., 2022, p. 1177).

### Implications for Further Research

We did not identify any study from LMICs or from the UK. Further research from qualitative research on the perspectives of people living with chronic conditions and their participation in leisure as well as a review of quantitative studies, particularly rehabilitation trials evaluating the effectiveness of occupational therapy interventions for promoting occupational engagement among these individuals are needed to inform practice. More perceptions from patients’ lived experiences would have been valuable for illuminating unexpected perspectives that proxies may not have revealed. Another important area for future research is the recruitment of vulnerable groups like the target population to ensure people are invited to participate in an ethical and person-centred way. In Peoples et al. (2017, p. 58) study, several participants declined participation; therefore, the findings should be interpreted with caution because it is difficult to determine whether these are truly representative of the full diversity of people living with advanced cancer.

To increase diversity, future studies should recruit a larger and less homogeneous sample, such as people from different ethnic backgrounds, and people from lower socioeconomic groups. This is significant because ‘78%’ of people receiving palliative care worldwide live in LMICs, and these studies were conducted primarily in high-income countries, ignoring a large population receiving palliative care (Bassah et al., 2023, p. 2). A steering group of palliative patients who are not study participants could assist in defining low-intensity leisure activities, which would be beneficial because cross-cultural definitions of leisure can be investigated to get a more complete picture of leisure research (Purrington & Hickerson, 2013).

### Implications for Practice

This review implies the importance for OTs to systematically develop and evaluate interventions for improving leisure participation among those surviving cancer and those requiring palliative care following chronic illnesses. Health professionals could also investigate how interventions can be provided in a way that least interferes with people’s daily occupational routines. Because wellbeing is dependent on whether people can engage in occupations or whether engagement has been disrupted by cancer treatment (la Cour et al., 2009). There is a clear implication for systematic development of holistic and inclusive interventions with the primary involvement of OTs to enable palliative patients to independently engage in occupations that are meaningful for them. Collaboration between professions is critical for educating the wider team on the unique contribution of OTs to palliative care. This could occur during discussions about improving palliative care services in which professionals focus on not only addressing symptoms but seek to build a deeper understanding of the ways in which people create meaning in their daily lives. This may shape the development of outpatient services for people with advanced cancer living at home.

### Implications for Policy

Policies should be developed to integrate palliative care services into the design and funding of all healthcare systems, as well as policies to strengthen and expand the workforce, such as training for existing health professionals to support their awareness and understanding of the occupational therapy role, incorporating palliative care into the core curricula of all new health professionals, and providing students with palliative care placements. This could be achieved through role-emerging placements.

## Conclusion

### Leisure Activities for People With Advanced Cancer Contribute to Health and Wellbeing

(Reynolds & Prior, 2006). However, cancer affects occupational performance as people find it difficult to participate in occupations when they feel unwell (Hammill et al., 2019, p.147). This review emphasised the importance of OTs working in palliative care using activity analysis to grade and adapt occupations and environments so that patients can participate in meaningful leisure. This review has revealed barriers impeding OTs’ ability to meet the occupational needs of patients, and more qualitative research is needed to gain a deeper understanding of the lived experiences of patients receiving palliative care and the value they place on leisure, as well as research into the developing roles for OTs in palliative care, and the effectiveness of occupation-focused practice.

**Key Findings**

- Leisure activities for people living with advanced cancer contribute to health and wellbeing.
- Cancer negatively affects occupational engagement and performance.
- Occupational therapy interventions can increase leisure engagement.

**What the Review has Added**

- This review has highlighted the value of leisure in palliative care, as well as the unique role of OTs in facilitating participation in leisure activities through systematic development and evaluation of holistic and inclusive interventions for improving occupational engagement among those affected by terminal illness.

## Supplemental Material

sj-docx-1-bjo-10.1177_03080226251367641 – Supplemental material for Interventions for Improving Leisure for Older Adults on the Palliative Pathway Living With Advanced Cancer: A Qualitative Systematic Review Supplemental material, sj-docx-1-bjo-10.1177_03080226251367641 for Interventions for Improving Leisure for Older Adults on the Palliative Pathway Living With Advanced Cancer: A Qualitative Systematic Review by Collette Crilly and Sureshkumar Kamalakannan in British Journal of Occupational Therapy

## References

[bibr1-03080226251367641] Age UK (2019) End of life care (England). Report, U.K. https://www.ageuk.org.uk/globalassets/age-uk/documents/policy-positions/care-and-support/ppp_end_of_life_care_en.pdf

[bibr2-03080226251367641] Al-Abdin A Hunter-Jones P Sudbury-Riley L , et al. (2020) Dying-well: The contribution of leisure services to hospice care. Annals of Leisure Research 24(3): 340–359.

[bibr3-03080226251367641] Badger S Macleod R Honey A (2016) ‘It’s not about treatment, it’s how to improve your life’: The lived experience of occupational therapy in palliative care. Palliative & Supportive Care 14(3): 225–231. 26073536 10.1017/S1478951515000826

[bibr4-03080226251367641] Bassah N Vaughn L Santos Salas A (2023) Nurse-led adult palliative care models in low- and middle-income countries: A scoping review. Journal of Advanced Nursing 79: 4112–4126. 36965072 10.1111/jan.15646

[bibr5-03080226251367641] Bentz HH Madsen SH Pilegaard MS , et al. (2021) Occupations creating joy for people living with advanced cancer: A qualitative descriptive study. British Journal of Occupational Therapy 85(3): 187–198. 40337079 10.1177/03080226211009419 PMC12033723

[bibr6-03080226251367641] Butler K Henry C (2011) The route to success in end-of-life care – achieving quality for occupational therapy. Report, NHS National End of Life Programme. https://www.england.nhs.uk/improvement-hub/wp-content/uploads/sites/44/2017/11/End-of-Life-Care-Route-to-Success-Occupational-Therapy.pdf

[bibr7-03080226251367641] Carter N Bryant-Lukosius D DiCenso A , et al. (2014) The use of triangulation in qualitative research. Oncology Nursing Forum 41(5): 545–547. 25158659 10.1188/14.ONF.545-547

[bibr8-03080226251367641] Department of Health. (2008). End of life care strategy – Promoting high quality care for all adults at the end of life.

[bibr9-03080226251367641] Dudley H Mutebi N (2022) Palliative and end of life care. The Parliamentary Office of Science and Technology.

[bibr10-03080226251367641] Gómez-Batiste X Murray SA Thomas K , et al. (2017) Comprehensive and integrated palliative care for people with advanced chronic conditions: An update from several European initiatives and recommendations for policy. Journal of Pain and Symptom Management 53(3): 509–517. 28042069 10.1016/j.jpainsymman.2016.10.361

[bibr11-03080226251367641] Hammill K Bye R Cook C (2014) Occupational therapy for people living with a life-limiting illness: A thematic review. British Journal of Occupational Therapy 77(11): 582–589. 10.1111/1440-1630.12557 30666645

[bibr12-03080226251367641] Hammill K Bye R Cook C (2019) Occupational engagement of people living with a life-limiting illness: Occupational therapists’ perceptions. Australian Occupational Therapy Journal 66(2): 145–153. 30666645 10.1111/1440-1630.12557

[bibr13-03080226251367641] Higgins JPT Thomas J Chandler J , et al. (Eds.) (2022). Cochrane Handbook for Systematic Reviews of Interventions version 6.3. [online] Cochrane. https://www.training.cochrane.org/handbook

[bibr14-03080226251367641] Hitch D Pépin G Stagnitti K (2014) In the footsteps of Wilcock, Part one: The evolution of doing, being, becoming, and belonging. Occupational Therapy in Health Care 28(3): 231–246. 24689506 10.3109/07380577.2014.898114

[bibr15-03080226251367641] Hong QN Fàbregues S Bartlett G , et al. (2021). The Mixed Methods Appraisal Tool (MMAT) version 2018 for information professionals and researchers. Education for Information 34(4): 285–291.

[bibr16-03080226251367641] Imanishi M Tomohisa H Higaki K (2015) In-home occupational therapy for a patient with stage IV lung cancer: Changes in quality of life and analysis of causes. SpringerPlus 4: 157. 25853034 10.1186/s40064-015-0931-9 PMC4382498

[bibr17-03080226251367641] Keesing S Rosenwax L (2011) Is occupation missing from occupational therapy in palliative care? Australian Occupational Therapy Journal 58(5): 329–336. 21957917 10.1111/j.1440-1630.2011.00958.x

[bibr18-03080226251367641] Kircher C Hanna T Tranmer J , et al. (2020) Defining and implementing early palliative care for persons diagnosed with a life-limiting chronic illness: A scoping review protocol. JBI Evidence Synthesis 18(11): 2335–2341. 10.11124/JBISRIR-D-19-00377 33181592

[bibr19-03080226251367641] Konno K Akasaka M Koshida C , et al. (2020) Ignoring non-English-language studies may bias ecological meta-analyses. Ecology and Evolution 10(13): 6373–6384. 32724519 10.1002/ece3.6368 PMC7381574

[bibr20-03080226251367641] la Cour K Nordel lK Josephsson S (2009) Everyday lives of people with advanced cancer: Activity, time, location, and experience. OTJR: Occupation, Participation and Health 29(4): 154–162.

[bibr21-03080226251367641] Lasserson TJ Thomas J Higgins JP (2023) Chapter 1: Starting a review. [online] The Cochrane Collaboration. In: Higgins JPT Thomas J Chandler J , et al. (eds) Cochrane handbook for systematic reviews of interventions version 6.5. https://training.cochrane.org/handbook/current/chapter-01

[bibr22-03080226251367641] Lyons KD Lambert LA Balan S , et al. (2013) Changes in activity levels of older adult cancer survivors. OTJR: Occupation, Participation and Health 33(1): 31–39.

[bibr23-03080226251367641] Methley AM Campbell S Chew-Graham C , et al. (2014) PICO, PICOS and SPIDER: A comparison study of specificity and sensitivity in three search tools for qualitative systematic reviews. BMC Health Services Research 14: 579. 25413154 10.1186/s12913-014-0579-0 PMC4310146

[bibr24-03080226251367641] Morgan E (2019) ‘Living longer and old-age dependency – What does the future hold?’ Office for National statsitics (ONS) U.K. https://www.ons.gov.uk/peoplepopulationandcommunity/birthsdeathsandmarriages/ageing/articles/livinglongerandoldagedependencywhatdoesthefuturehold/2019-06-24

[bibr25-03080226251367641] National Cancer Institute (2021) Palliative care in cancer. National Cancer Institute. National Academies Press (US). https://www.cancer.gov/about-cancer/advanced-cancer/care-choices/palliative-care-fact-sheet

[bibr26-03080226251367641] National Cancer Institute (2023) NCI dictionary of cancer terms. National Academies Press (US). https://www.cancer.gov/publications/dictionaries/cancer-terms/expand/A#

[bibr27-03080226251367641] Noble H Smith J (2015) Issues of validity and reliability in qualitative research. Evidence-Based Nursing 18(2): 34–35. 25653237 10.1136/eb-2015-102054

[bibr28-03080226251367641] Nowell LS Norris JM White DE , et al. (2017) Thematic analysis: Striving to meet the trustworthiness criteria. International Journal of Qualitative Methods 16(1): 160940691773384.

[bibr29-03080226251367641] Olmos-Vega FM Stalmeijer RE Varpio L , et al. (2023) A practical guide to reflexivity in qualitative research: AMEE Guide No. 149. Medical Teacher 45(3): 241–251. 10.1080/0142159X.2022.2057287 35389310

[bibr30-03080226251367641] Page MJ McKenzie JE Bossuyt PM , et al. (2021) The PRISMA 2020 statement: An updated guideline for reporting systematic reviews. BMJ 372: n71. 10.1136/bmj.n71 PMC8005924 33782057

[bibr31-03080226251367641] Peoples H Brandt Å Wæhrens EE , et al. (2017) Managing occupations in everyday life for people with advanced cancer living at home. Scandinavian Journal of Occupational Therapy 24(1): 57–64. 27578556 10.1080/11038128.2016.1225815

[bibr32-03080226251367641] Purrington A Hickerson B (2013) Leisure as a cross-cultural concept. World Leisure Journal 55(2): 25–137.

[bibr33-03080226251367641] Reynolds F Prior S (2006) Creative adventures and flow in artmaking: A qualitative study of women living with cancer. British Journal of Occupational Therapy 69(6): 1–8.

[bibr34-03080226251367641] Rome RB Luminais HH Bourgeois DA , et al. (2011) The role of palliative care at the end of life. The Ochsner Journal 11(4): 348–352. 22190887 PMC3241069

[bibr35-03080226251367641] Shallwani SM Thomas R King J , et al. (2023) Perspectives and experiences of leisure-time physical activity in adults with stage 4 cancer: A qualitative interpretive-description study. Disability and Rehabilitation 46(8): 1515–1526. 37067063 10.1080/09638288.2023.2200037

[bibr36-03080226251367641] Shringarpure S Xing EP (2014) Effects of sample selection bias on the accuracy of population structure and ancestry inference. G3 (Bethesda, Md.) 4(5): 901–911. 24637351 10.1534/g3.113.007633 PMC4025489

[bibr37-03080226251367641] Soilemezi D Linceviciute S (2018) Synthesizing qualitative research. International Journal of Qualitative Methods 17(1): 1–14.

[bibr38-03080226251367641] Talbot-Coulombe C Bravo G Carrier A (2022) Occupational therapy practice in palliative and end-of-life care in Québec. Canadian Journal of Occupational Therapy. Revue canadienne d’ergotherapie 89(2): 201–211. 10.1177/00084174221084466 PMC9136369 35243918

[bibr39-03080226251367641] Tavemark S Hermansson LN Blomberg K (2019) Enabling activity in palliative care: Focus groups among occupational therapists. BMC Palliative Care 8(1): 17. 10.1186/s12904-019-0394-9 PMC6367774 30732615

[bibr40-03080226251367641] University of Canberra (2022) Systematic reviews in health. https://canberra.libguides.com/systematic/extract

[bibr41-03080226251367641] University of Michigan Library (2023) Systematic reviews. https://guides.lib.umich.edu/c.php?g=283340&p=2126706#s-lg-box-6479850

[bibr42-03080226251367641] Wasti SP Simkhada P van Teijlingen ER , et al. (2022) The growing importance of mixed-methods research in health. Nepal Journal of Epidemiology 12(1): 1175–1178. 35528457 10.3126/nje.v12i1.43633 PMC9057171

[bibr43-03080226251367641] World Health Organization (2020) Palliative Care. World Health Organization (WHO). https://www.who.int/news-room/fact-sheets/detail/palliative-care

