# Supplementary material for: Interventions for Improving Leisure for Older Adults on the Palliative Pathway Living With Advanced Cancer: A Qualitative Systematic Review
Source: Br J Occup Ther. 2025 Oct 27;89(5):297–310. doi: 10.1177/03080226251367641 (PMC13133478; doi:10.1177/03080226251367641)
Supplement: sj-docx-1-bjo-10.1177_03080226251367641 – Supplemental material for Interventions for Improving Leisure for Older Adults on the Palliative Pathway Living With Advanced Cancer: A Qualitative Systematic Review [file sj-docx-1-bjo-10.1177_03080226251367641.docx]

# Appendices

**Appendix 1 - Cochrane Data Extraction Form to guide Pro Forma** (Higgins et al., 2022)


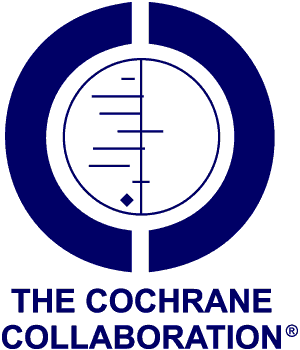
Data collection form

Intervention review – RCTs and non-RCTs

This form can be used as a guide for developing your own data extraction form. Sections can be expanded and added, and irrelevant sections can be removed. It is difficult to design a single form that meets the needs of all reviews, so it is important to consider carefully the information you need to collect, and design your form accordingly. Information included on this form should be comprehensive, and may be used in the text of your review, ‘Characteristics of included studies’ table, risk of bias assessment, and statistical analysis.

## Notes on using data extraction form:

- Be consistent in the order and style you use to describe the information for each report.
- Record any missing information as unclear or not described, to make it clear that the information was not found in the study report(s), not that you forgot to extract it.
- Include any instructions and decision rules on the data collection form, or in an accompanying document. It is important to practice using the form and give training to any other authors using the form.

| Review title or ID |  |
| --- | --- |
| Study ID *(surname of first author and year first full report of study was published e.g. Smith 2001)* |  |
| Report ID |  |
| Report ID of other reports of this study |  |
| Notes | |

# General Information

| Date form completed *(dd/mm/yyyy)* |  |
| --- | --- |
| Name/ID of person extracting data |  |
| Reference citation |  |
| Study author contact details |  |
| Publication type *(e.g. full report, abstract, letter)* |  |
| Notes: | |

# Study eligibility

| Study Characteristics | Eligibility criteria  *(Insert inclusion criteria for each characteristic as defined in the Protocol)* | | Eligibility criteria met? | | | Location in text or source *(pg & ¶/fig/table/other)* |
| --- | --- | --- | --- | --- | --- | --- |
|  |  | | Yes | No | Unclear |  |
| Type of study | Randomised Controlled Trial | |  |  |  |  |
|  | Quasi-randomised Controlled Trial | |  |  |  |  |
|  | Controlled Before and After Study  Contemporaneous data collection  Comparable control sites  At least 2 x intervention and 2 x control clusters | |  |  |  |  |
|  | Interrupted Time Series  At least 3 time points before and 3 after the intervention  Clearly defined intervention point | |  |  |  |  |
|  | Other design (specify): | |  |  |  |  |
| Participants |  | |  |  |  |  |
| Types of intervention |  | |  |  |  |  |
| Types of comparison |  | |  |  |  |  |
| Types of outcome measures |  | |  |  |  |  |
| INCLUDE | | EXCLUDE | | | | |
| Reason for exclusion |  | | | | | |
| Notes: | | | | | | |

**DO NOT PROCEED IF STUDY EXCLUDED FROM REVIEW**

# Characteristics of included studies

## Methods

|  | **Descriptions as stated in report/paper** | | **Location in text or source** *(pg & ¶/fig/table/other)* |
| --- | --- | --- | --- |
| **Aim of study** *(e.g. efficacy, equivalence, pragmatic)* |  | |  |
| **Design***(e.g. parallel, crossover, non-RCT)* |  | |  |
| **Unit of allocation**  *(by individuals, cluster/ groups or body parts)* |  | |  |
| **Start date** |  | |  |
| **End date** |  | |  |
| **Duration of participation**  *(from recruitment to last follow-up)* |  | |  |
| **Ethical approval needed/ obtained for study** | Yes No Unclear |  |  |
| **Notes:** | | | |

## Participants

|  | Description  *Include comparative information for each intervention or comparison group if available* | | Location in text or source *(pg & ¶/fig/table/other)* |
| --- | --- | --- | --- |
| Population description  *(from which study participants are drawn)* |  | |  |
| Setting  *(including location and social context)* |  | |  |
| Inclusion criteria |  | |  |
| Exclusion criteria |  | |  |
| Method of recruitment of participants *(e.g. phone, mail, clinic patients)* |  | |  |
| Informed consent obtained | Yes No Unclear |  |  |
| Total no. randomised  *(or total pop. at start of study for NRCTs)* |  | |  |
| Clusters  *(if applicable, no., type, no. people per cluster)* |  | |  |
| Baseline imbalances |  | |  |
| Withdrawals and exclusions  *(if not provided below by outcome)* |  | |  |
| Age |  | |  |
| Sex |  | |  |
| Race/Ethnicity |  | |  |
| Severity of illness |  | |  |
| Co-morbidities |  | |  |
| Other relevant sociodemographics |  | |  |
| Subgroups measure |  | |  |
| Subgroups reported |  | |  |
| Notes: | | | |

## Intervention groups

*Copy and paste table for each intervention and comparison group*

**Intervention Group 1**

|  | Description as stated in report/paper | Location in text or source *(pg & ¶/fig/table/other)* |
| --- | --- | --- |
| Group name |  |  |
| No. randomised to group  *(specify whether no. people or clusters)* |  |  |
| Theoretical basis *(include key references)* |  |  |
| Description *(include sufficient detail for replication, e.g. content, dose, components)* |  |  |
| Duration of treatment period |  |  |
| Timing *(e.g. frequency, duration of each episode)* |  |  |
| Delivery *(e.g. mechanism, medium, intensity, fidelity)* |  |  |
| Providers  *(e.g. no., profession, training, ethnicity etc. if relevant)* |  |  |
| Co-interventions |  |  |
| Economic information *(i.e. intervention cost, changes in other costs as result of intervention)* |  |  |
| Resource requirements  *(e.g. staff numbers, cold chain, equipment)* |  |  |
| Integrity of delivery |  |  |
| Compliance |  |  |
| Notes: | | |

## Outcomes

*Copy and paste table for each outcome.*

**Outcome 1**

|  | Description as stated in report/paper | | Location in text or source *(pg & ¶/fig/table/other)* |
| --- | --- | --- | --- |
| Outcome name |  | |  |
| Time points measured  *(specify whether from start or end of intervention)* |  | |  |
| Time points reported |  | |  |
| Outcome definition *(with diagnostic criteria if relevant)* |  | |  |
| Person measuring/ reporting |  | |  |
| Unit of measurement  *(if relevant)* |  | |  |
| Scales: upper and lower limits *(indicate whether high or low score is good)* |  | |  |
| Is outcome/tool validated? | Yes No Unclear |  |  |
| Imputation of missing data *(e.g. assumptions made for ITT analysis)* |  | |  |
| Assumed risk estimate  *(e.g. baseline or population risk noted in Background)* |  | |  |
| Power *(e.g. power & sample size calculation, level of power achieved)* |  | |  |
| Notes: | | | |

## Other

| **Study funding sources** *(including role of funders)* |  |  |
| --- | --- | --- |
| **Possible conflicts of interest** *(for study authors)* |  |  |
| **Notes:** | | |

# Risk of Bias assessment

*See* [*Chapter 8*](http://www.mrc-bsu.cam.ac.uk/cochrane/handbook/index.htm#chapter_8/8_assessing_risk_of_bias_in_included_studies.htm) *of the Cochrane Handbook. Additional domains may be added for non-randomised studies.*

| Domain | Risk of bias | | | Support for judgement  *(include direct quotes where available with explanatory comments)* | Location in text or source *(pg & ¶/fig/table/other)* |
| --- | --- | --- | --- | --- | --- |
|  | Low | High | Unclear |  |  |
| Random sequence generation  *(selection bias)* |  |  |  |  |  |
| Allocation concealment  *(selection bias)* |  |  |  |  |  |
| Blinding of participants and personnel  *(performance bias)* |  |  |  | Outcome group: All/ |  |
| *(if separate judgement by outcome(s) required)* |  |  |  | Outcome group: |  |
| Blinding of outcome assessment  *(detection bias)* |  |  |  | Outcome group: All/ |  |
| *(if separate judgement by outcome(s) required)* |  |  |  | Outcome group: |  |
| Incomplete outcome data  *(attrition bias)* |  |  |  | Outcome group: All/ |  |
| *(if separate judgement by outcome(s) required)* |  |  |  | Outcome group: |  |
| Selective outcome reporting?  *(reporting bias)* |  |  |  |  |  |
| Other bias |  |  |  |  |  |
| Notes: | | | | | |

# Appendix 2 – Data Extraction Table Template


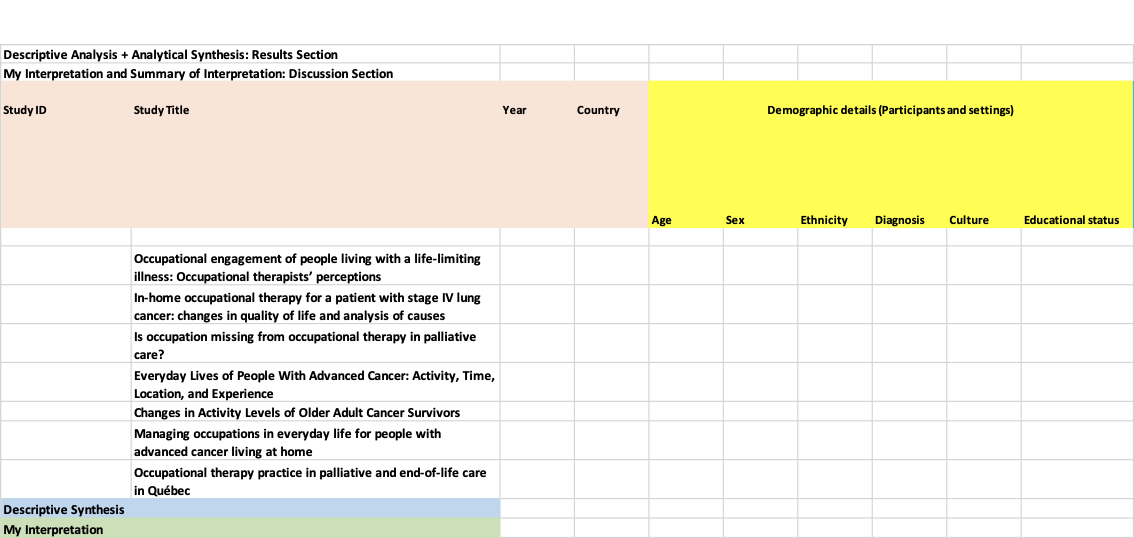


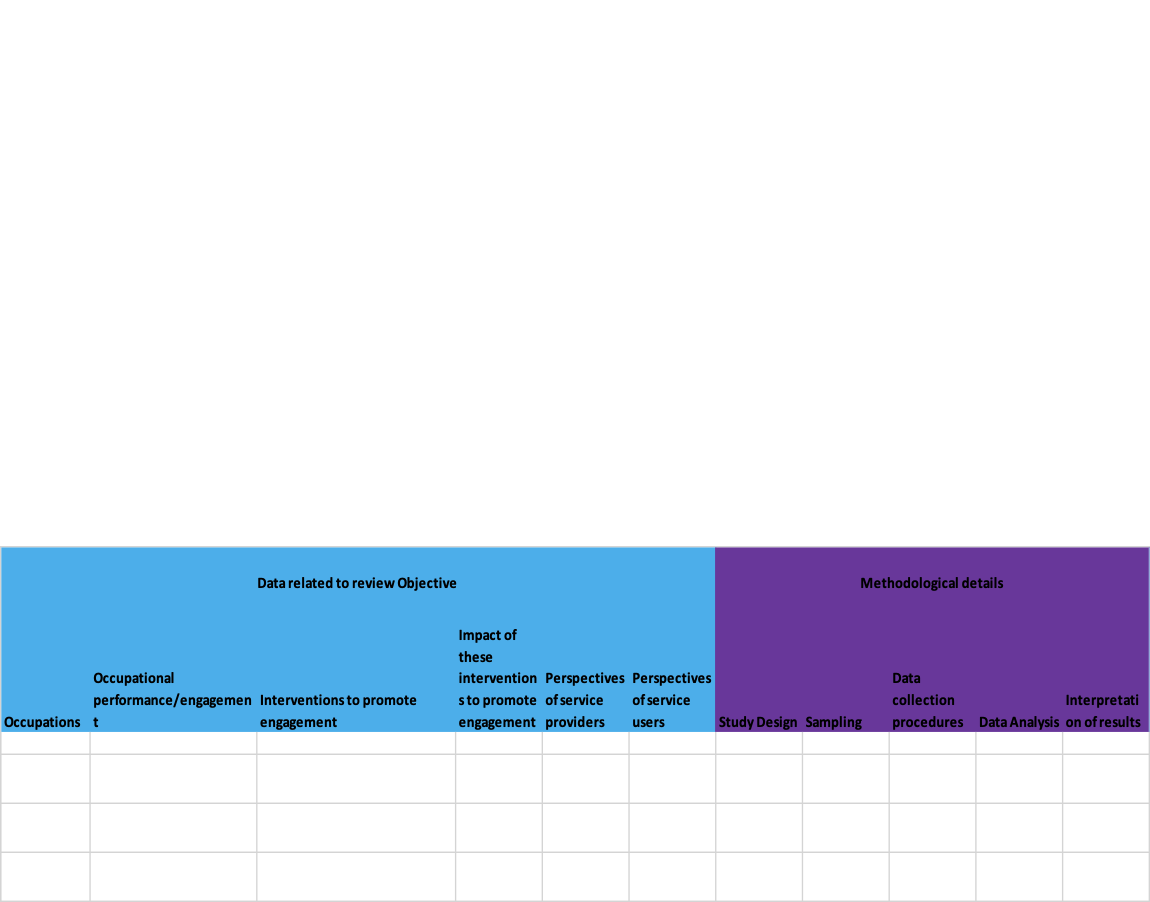


**Appendix 2 - Data Extraction Table Template (continued)**

**Appendix 3 – Stages of Thematic Analysis** (Nowell et al., 2017, p.4)

**Appendix 4 – The Mixed Methods Appraisal Tool Version 2018**

**
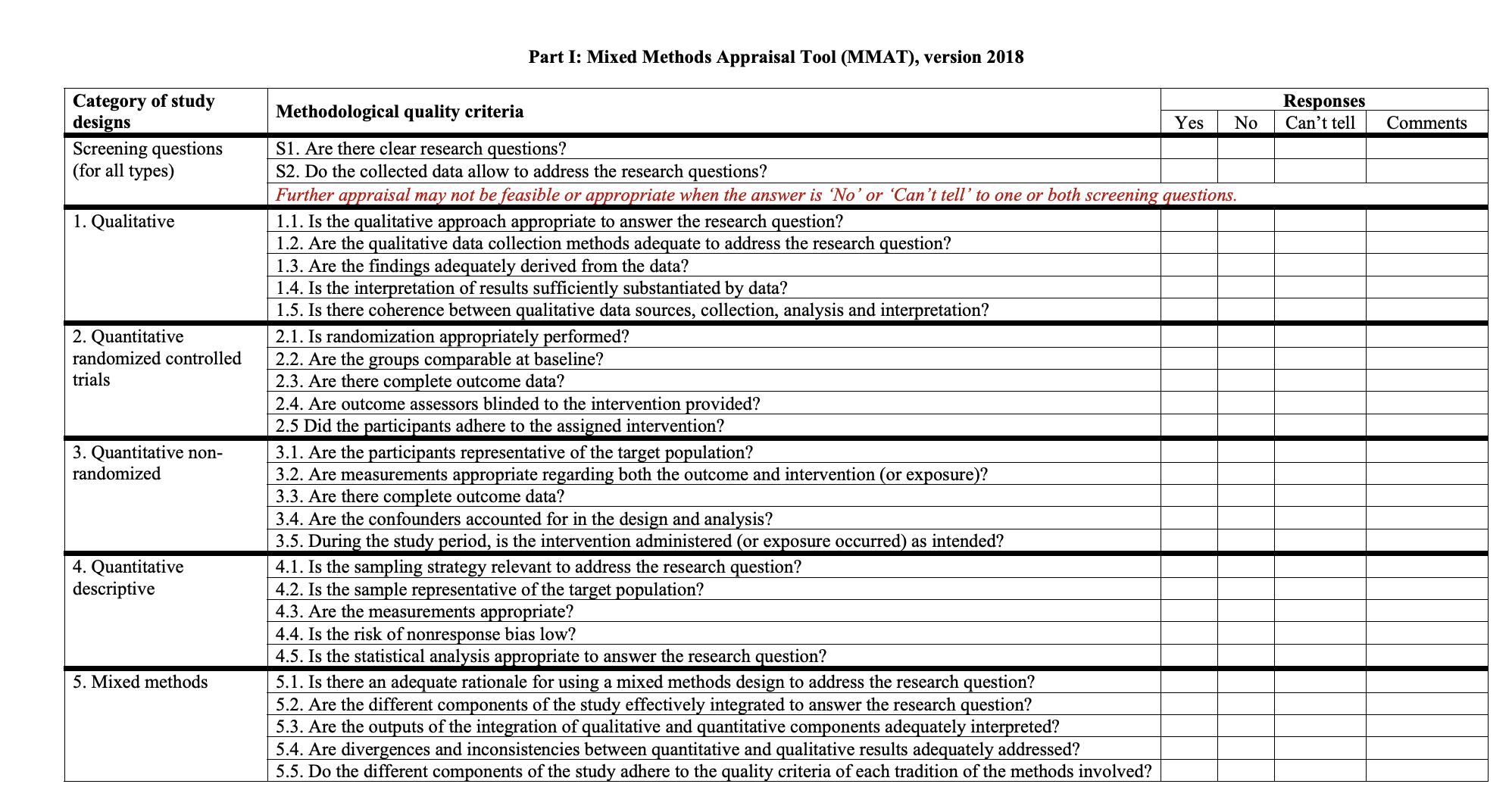
**

**Appendix 5 – Stages of Thematic Analysis**

*Phase 1: Familiarisation with collected data*

| **Data Collection** | **Keywords/Phrases identified** |
| --- | --- |
| In-home occupational therapy for a patient with stage IV lung cancer: changes in quality of life and analysis of causes | “Pain”  “Despair”  “Anger”  “Impatience”  “Loneliness hampers recovery”  “Gratitude for being able to cook and eat independently”  “Occupational therapy provided acceptance of death and a sense of peace and hope”. |
| Is occupation missing from occupational therapy in palliative care? | “Couldn’t go fishing”  “Confined to the home”  “I can’t do anything for myself, I’m hopeless”  “Might have been useful if he’d had a support group”  “Helpful to talk to men on the same ward that go through similar things”  “Driving was another big loss”  “OT service is very reactive, just discharge planning, crisis management”  “Not doing a lot at the moment as there just isn’t the capacity to do it”  “Equipment provider”  “Discharge planner”  “The pressure is that you’ve got fifteen patients on your team you’ve got to get them out within two days”  “Not a lot of time to do what OTs should be doing”  “We know we can do so much more and we need to be looking at the whole individual and we don’t have the resources to do it” |
| Everyday Lives of People With Advanced Cancer: Activity, Time, Location, and Experience | “House hold activity and gardening are enriching”  “I have lots of things I can occupy myself with but my need is also to get together with others around craftwork, something practical like gardening”  “I cannot do much with my back”  “days are pretty much like always” “familiar”  “I do the things I have always done – read the newspaper and watch TV. I have always liked to follow what goes on in society”  “It is important for me to have something to attend to, I need to do something every day so that I have what is almost a working day”.  “I have rhythms for everything right from when I get up in the morning at 6:50”  “In some ways my life is very monotonous, the days repeat themselves, but I still find that things happen that are different”.  “Fiddling, sanding, exchanging ideas of how best to fix it, it is the process of thinking in entirely different ways and watching it grow”.  “I was annoyed at the conversation with the doctor about whether I would rather take my medication as pills. I was not interested. It does not fit into my day”.  “I would like them to have brought us together. I actually think there is something (an activity) Friday afternoons at the hospital”.  “There are days when I cannot even mow the lawn then; I get out of breath. But I can at least sit outside and watch the animals”  “There are days when you are not well and then you just stay at home”. |
| Changes in Activity Levels of Older Adult Cancer Survivors | “Spectator sports”  “Recreational shopping”  “Table games”  “Puzzles”  “Drawing”  “Reading magazines”  “Creative writing”  “Bird watching”  “Watching TV”  “Family gatherings”  “Talking on telephone”  “I can’t travel like I did before and I travelled a lot”  “There are friends that I didn’t see a whole lot before because I travelled so much, that have come into my life in a very different way. So I’m seeing more of them, and that is the balancing bit”  “Anything that has gone to a less frequent activity is a result of my being tired, a natural consequence of the kind of surgery I had”  “You’re just out of gas”  “I seem to tire easily”  “I felt the need to keep my life as normal as I could keep it, and I think it saved me”.  “I may have to make adjustments in terms of the- either the extent of what I used to do, or the amount of what I used to do, or the way I used to do it before”.  “I still want to be a part of my world, and I still want to partake of that”.  “Cut back on but not cut it out totally”. |
| Managing occupations in everyday life for people with advanced cancer living at home | “My life changed from black to white”  “I used to go to work and was active around the house and the garden”  “Now I’m lucky if I have two-five minutes to do something and then I’m finished”  “It used to be me who made the breakfast every morning, something I have done since we got married, now it’s my wife who has to make it”.  “I have a very boring life”  “I thought my god this is sad”  “All my daily activities are completely gone and it is a loss”  “I do one thing and then take a break”  “There are days I exceed my limit and then I collapse and lie in bed for three days”  “I’m no longer able to drive, I order my groceries online”  “Those are the things I’m focused on right now and when I’ve achieved them I can take the next step”  “I now use a rollator when I go to the supermarket” |
| Occupational therapy practice in palliative and end-of-life care in Québec | “Ensure that the person and staff have taken all possible measures to limit the risk of wounds/minimise the wound”.  “Adapt the activity or environment to allow the person to perform the activity he or she considers significant”  “Evaluation of needs in terms of positioning in the room, but also for hospital departure if necessary”  “According to the client’s wishes, certain important activities are targeted, often hygiene and using the toilet”  “Since it’s not always easy to get the equipment quickly clients sometimes stay in hospital much longer than expected”  “I have to have a waiting list; it puts a lot of pressure on me”  “I have trouble doing meaningful follow-ups because I don’t have the time”  “Other professionals do not understand the role of occupational therapists”  “Equipment delivery person”  “Referrals by other team members are too specific rather than letting us do our own analyses” |
| Occupational engagement of people living with a life-limiting illness: Occupational therapists’ perceptions | “Focusing on life”  “Preparing for death”  “Engaging in domestic routines”  “Spending time with close family and friends”  “Still felt alive and not like they are waiting to die”  “I think it is the way the person prioritises their occupations”  “Goals at this time are more based around leisure and social performance as opposed to self-care and productivity domains”  “Big focus on social participation when clients have less physical abilities”  “Spend quiet time gathering the person’s narrative and facilitating problem-solving to allow people to engage in meaningful occupations in their most natural environment”  “It may be the way that occupations are done that is unique based on the person’s abilities, and comfort and level of assistance needed”  “Maintains dignity”  “Reduce stress”  “I think people at the end of life want to engage in being, thinking, and processing”  “Singing if possible, dancing if possible. If not watching others dance/perform in traditional ceremonies”  “Get more out of what they wanted to do”  “Completion of tasks, having affairs in order such as writing wills and organising finances”  “Sense of completion”  “Facilitate closure” |

*Phase 2: Generating initial codes*

| **Data Extract** | **Codes Identified** |
| --- | --- |
| “House hold activity and gardening are enriching”  “I do the things I have always done – read the newspaper and watch TV. I have always liked to follow what goes on in society”  “Spectator sports”  “Recreational shopping”  “Table games”  “Puzzles”  “Drawing”  “Reading magazines”  “Creative writing”  “Bird watching”  “Watching TV”  “Family gatherings”  “Talking on telephone”  “Completion of tasks, having affairs in order such as writing wills and organising finances”  “Sense of completion”  “Facilitate closure”  “Engaging in domestic routines” | Examples of occupations  Leisure activities  Low-intensity leisure |
| “Pain”  “Despair”  “Anger”  “Impatience”  “Loneliness hampers recovery”  Couldn’t go fishing”  “Confined to the home”  “There are days when I cannot even mow the lawn then; I get out of breath. But I can at least sit outside and watch the animals”  “There are days when you are not well and then you just stay at home”.  “I can’t travel like I did before and I travelled a lot”  “There are friends that I didn’t see a whole lot before because I travelled so much, that have come into my life in a very different way. So I’m seeing more of them, and that is the balancing bit”  “Anything that has gone to a less frequent activity is a result of my being tired, a natural consequence of the kind of surgery I had”  “You’re just out of gas”  “I seem to tire easily”  “My life changed from black to white”  “I used to go to work and was active around the house and the garden”  “Now I’m lucky if I have two-five minutes to do something and then I’m finished”  “It used to be me who made the breakfast every morning, something I have done since we got married, now it’s my wife who has to make it”.  “I have a very boring life”  “I thought my god this is sad”  “All my daily activities are completely gone and it is a loss” | Effects of cancer on engagement and performance  Decreased engagement  Decreased performance  Increased engagement |
| “Might have been useful if he’d had a support group”  “Helpful to talk to men on the same ward that go through similar things”  “I have lots of things I can occupy myself with but my need is also to get together with others around craftwork, something practical like gardening”  “I would like them to have brought us together. I actually think there is something (an activity) Friday afternoons at the hospital”.  “There are days when I cannot even mow the lawn then; I get out of breath. But I can at least sit outside and watch the animals”  “I felt the need to keep my life as normal as I could keep it, and I think it saved me”.  “I may have to make adjustments in terms of the- either the extent of what I used to do, or the amount of what I used to do, or the way I used to do it before”.  “I still want to be a part of my world, and I still want to partake of that”.  My life changed from black to white”  “I used to go to work and was active around the house and the garden”  “Now I’m lucky if I have two-five minutes to do something and then I’m finished”  “I do one thing and then take a break”  “There are days I exceed my limit and then I collapse and lie in bed for three days”  “I’m no longer able to drive, I order my groceries online”  “Those are the things I’m focused on right now and when I’ve achieved them I can take the next step”  “I now use a rollator when I go to the supermarket”  “It may be the way that occupations are done that is unique based on the person’s abilities, and comfort and level of assistance needed”  “I think people at the end of life want to engage in being, thinking, and processing”  “Singing if possible, dancing if possible. If not watching others dance/perform in traditional ceremonies”  “Get more out of what they wanted to do” | Grading and adapting  Social participation  Peer support groups  Equipment provision  Refocusing occupations  Pacing techniques |
| “Gratitude for being able to cook and eat independently”  “Occupational therapy provided acceptance of death and a sense of peace and hope”.  “It is important for me to have something to attend to, I need to do something every day so that I have what is almost a working day”.  “Fiddling, sanding, exchanging ideas of how best to fix it, it is the process of thinking in entirely different ways and watching it grow”.  “Still felt alive and not like they are waiting to die”  “Maintains dignity”  “Reduce stress”  “Sense of completion”  “Facilitate closure” | Normality  Familiarity and routines  Maintaining independence  Positive feelings of hope and peace  Creative challenge and a sense of flow  Focus on living  Maintain sense of identity  Acceptance of death |
| “OT service is very reactive, just discharge planning, crisis management”  “Not doing a lot at the moment as there just isn’t the capacity to do it”  “Equipment provider”  “Discharge planner”  “The pressure is that you’ve got fifteen patients on your team you’ve got to get them out within two days”  “Not a lot of time to do what OTs should be doing”  “We know we can do so much more and we need to be looking at the whole individual and we don’t have the resources to do it”  Since it’s not always easy to get the equipment quickly clients sometimes stay in hospital much longer than expected”  “I have to have a waiting list; it puts a lot of pressure on me”  “I have trouble doing meaningful follow-ups because I don’t have the time”  “Other professionals do not understand the role of occupational therapists”  “Equipment delivery person”  “Referrals by other team members are too specific rather than letting us do our own analyses”  “Goals at this time are more based around leisure and social performance as opposed to self-care and productivity domains”  “Big focus on social participation when clients have less physical abilities”  “Spend quiet time gathering the person’s narrative and facilitating problem-solving to allow people to engage in meaningful occupations in their most natural environment” | Barriers to occupational therapy practice  Ability to meet people’s occupational needs such as leisure  Reframing engagement from ‘doing’ to ‘being’  Misunderstanding of the occupational therapy role |
| “I was annoyed at the conversation with the doctor about whether I would rather take my medication as pills. I was not interested. It does not fit into my day”.  “I would like them to have brought us together. I actually think there is something (an activity) Friday afternoons at the hospital”.  My life changed from black to white”  “I used to go to work and was active around the house and the garden”  “Now I’m lucky if I have two-five minutes to do something and then I’m finished”  “It used to be me who made the breakfast every morning, something I have done since we got married, now it’s my wife who has to make it”.  “I have a very boring life”  “I thought my god this is sad”  “All my daily activities are completely gone and it is a loss”  “Focusing on life”  “Preparing for death”  “Engaging in domestic routines”  “Spending time with close family and friends” | Focusing on living  Preparing for death  Social participation is important  Negative feelings associated with loss  Reflection on life before cancer  Disruption to routines |

*Phase 3: Searching for themes*

| **Codes Identified** | **Themes** |
| --- | --- |
| Examples of occupations  Leisure activities  Low-intensity leisure | **Occupations** |
| Effects of cancer on engagement and performance  Decreased engagement  Decreased performance  Increased engagement | **Occupational performance and engagement** |
| Grading and adapting  Social participation  Peer support groups  Equipment provision  Refocusing occupations  Pacing techniques | **Interventions to promote engagement** |
| Normality  Familiarity and routines  Maintaining independence  Positive feelings of hope and peace  Creative challenge and a sense of flow  Focus on living  Maintain sense of identity  Acceptance of death | **Impact of interventions to promote engagement** |
| Barriers to occupational therapy practice  Ability to meet people’s occupational needs such as leisure  Reframing engagement from ‘doing’ to ‘being’  Misunderstanding of the occupational therapy role | **Perspectives of occupational therapists** |
| Focusing on living  Preparing for death  Social participation is important  Negative feelings associated with loss  Reflection on life before cancer  Disruption to routines | **Perspectives of patients** |

*Phase 4: Review themes*

| **Final themes** | **Discarded themes** |
| --- | --- |
| Occupations | None |
| Occupational performance and engagement |  |
| Interventions to promote engagement |  |
| Impact of interventions to promote engagement |  |
| Perspectives of occupational therapists |  |
| Perspectives of patients |  |
